# Fabrication of a Novel and Ultrasensitive Label-Free Electrochemical Aptasensor Based on Gold Nanostructure for Detection of Homocysteine

**DOI:** 10.3390/bios13020244

**Published:** 2023-02-08

**Authors:** Reza Zaimbashi, Somayeh Tajik, Hadi Beitollahi, Masoud Torkzadeh-Mahani

**Affiliations:** 1 Environment Department, Institute of Science and High Technology and Environmental Sciences, Graduate University of Advanced Technology, Kerman 7631818356, Iran; 2 Research Center of Tropical and Infectious Diseases, Kerman University of Medical Sciences, Kerman 7616913555, Iran; 3 Biotechnology Department, Institute of Science and High Technology and Environmental Sciences, Graduate University of Advanced Technology, Kerman 7631818356, Iran

**Keywords:** aptasensor, homocysteine, Au nanostructured, differential pulse voltammetry, carbon paste electrode

## Abstract

The current attempt was made to detect the amino acid homocysteine (HMC) using an electrochemical aptasensor. A high-specificity HMC aptamer was used to fabricate an Au nanostructured/carbon paste electrode (Au-NS/CPE). HMC at high blood concentration (hyperhomocysteinemia) can be associated with endothelial cell damage leading to blood vessel inflammation, thereby possibly resulting in atherogenesis leading to ischemic damage. Our proposed protocol was to selectively immobilize the aptamer on the gate electrode with a high affinity to the HMC. The absence of a clear alteration in the current due to common interferants (methionine (Met) and cysteine (Cys)) indicated the high specificity of the sensor. The aptasensor was successful in sensing HMC ranging between 0.1 and 30 μM, with a narrow limit of detection (LOD) as low as 0.03 μM.

## 1. Introduction

Homocysteine, 2-amino-4-sulfanylbutanoic acid, or HMC is found in human blood as an essential amino acid that is not introduced via food sources but originates from methionine, where the Met present in food is converted to cysteine (Cys) through homocysteine (HMC). The reaction can reverse from HMC to Met; if the reaction stops at HMC, it cannot proceed to Cys and Met, so that the HMC concentration in biological systems may rise above the normal dose of 5–16 μM, which is called hyperhomocysteinemia. As a result, this phenomenon can be associated with many health risks, such as heart attack, osteoporosis, and pregnancy complications [1,2,3,4].

Various techniques were previously used to quantify HMC, some of which are HPLC, GC-MS, capillary electrophoresis, two-photon fluorescent chemosensors, LC-ESI-MS/MS, and LC/MS/MS [5,6,7,8,9,10]. This issue highlights the importance of biomolecule measurement. Therefore, much attention has been drawn to sensors and biosensors based on electrochemical approaches with praiseworthy features in terms of selectivity, sensitivity, portability, and reliability [11,12,13,14,15,16,17,18,19,20,21,22,23,24,25,26,27,28,29,30,31,32,33,34,35,36,37,38]. Accordingly, it seems necessary to develop biosensors for quantitative determination of HMC in terms of avoiding health conditions.

There is evidence for the use of electrochemical approaches to determine HMC [39,40]. The result of such research in the last decade has been to reach an electrochemical detection platform based on aptamers (aptasensors). Reportedly, such a sensing system is an approach for the analysis of water and food specimens due to merits like low cost, green nature, and ease of use. For multiple electrochemical aptasensors, a structural alteration in the immobilized aptamer can occur due to interaction with the desired analytes, which changes the resistance of electron transfer between the electrode surface and redox active species. The sensors based on the electrochemical aptamer (EA), according to the molecular pathway of target-induced strand displacement (TISD), exhibit facile and effective systems in electrochemical platforms. The detection analytes used by such TISD-supported EA sensors are duplex probes consisting of electrode-immobilized aptamer-cDNA matrices. Hybridization of an aptamer sequence with its complementary DNA (cDNA) was engineered to obtain a duplex probe. When target molecules are present, the aptamer forms an aptamer-target complex, and the complementary strand of the aptamer-cDNA duplex is displaced, resulting in the TISD reaction at the sensor interface. On the one hand, it is possible to engineer duplex aptamer-cDNA probes based on facile base-pairing protocols for all aptamers. On the other hand, TISD detection can follow diverse electrochemical approaches for signal transduction. Therefore, TISD-supported EA sensors are developed to detect various targets, such as proteins, metal ions, and small organic substances [41,42,43,44,45,46].

The present attempt was made to integrate the merits of the above-mentioned substances and approaches to construct ultra-sensitive electrochemical sensors based on the modification of gold nanoparticles/carbon paste electrode surfaces with aptamers (AP/Au-NS/CPE). Based on the evidence, a facile fabrication of AP/Au-NS/CPE was successful in electrochemically biosensing the HMC. The suggested system is appreciable and reliable for electrochemically biosensing the HMC.

## 2. Experimental Procedure

### 2.1. Chemicals

The sequences of utilized oligonucleotides (Bio Basic Inc., Markham, ON, Canada) consisted of DNA, 5′SH-(CH_2_)_6_ ACCA GCAC ATTC GATT ATAC CAGC TTAT TCAA TTCA CAGC TATG TCCT ATAC CAGC TTAT TCAATT−3′ [47]. Citric acid, sodium hydroxide, acetic acid, phosphoric acid, acetone, and ethanol belonged to Merck. The 6-mercapto 1-hexanol and DL-homocysteine were from Sigma-Aldrich. The remaining chemicals were of reagent grade. 

### 2.2. Equipment 

The voltammetric analysis was performed by PGSTAT 302N Autolab potentiostat/galvanostat (the Netherlands). The conventional three-cell electrode system used contained a CPE work electrode, an Ag/AgCl reference electrode, and a platinum wire counter electrode. A Metrohm 713 pH meter was employed to measure pH values using a glass-reference electrode. To do DPVs, the CPE was placed in a constant concentration of HMC (5 mL) and 0.1 M PBS (0.1 M Na_2_HPO_4_–NaH_2_PO_4_ embarrassing 0.1 M NaCl, pH = 7.0) while stirring at ambient conditions. Following the PBS washing process, the CPE was positioned in a three-electrode system. The DPV determinations were performed in 0.1 M PBS (25 mL, pH 7.0) at ambient conditions. 

### 2.3. Fabrication of CPE

To construct the CPE, graphite powder (0.5 g) was blended with nujol oil (0.3 mL) in a glassy mortar. After preparing the carbon paste, it was placed inside the electrode hole and smoothed with a filter paper to obtain a shiny appearance. 

### 2.4. Modification of CPE Surface 

The CPE surface was modified with Au-NS by immersing the electrode inside the gold solution (6 mM AuCl_4_ + 0.1 M KNO_3_) at −400 mV for 400 s (Au-NS/CPE). The Au-NS/CPE surface was coated with a 4.5 μM aptamer solution (5 μL). The electrode was subsequently kept vertically in a moist chamber overnight for self-assembly. As-prepared Au-NS/CPE was positioned in 1 mM 6-mercapto 1-hexanol within 60 min after washing with 0.1 M PBS (pH = 7.0) to limit common sites and to obtain the aptamer strands in straight coordination. The washing process for the electrode in each step was performed via PBS (25 mM, pH = 7.0). 

### 2.5. Construction of Electrochemical Aptasensor 

The label-free aptasensor developed to detect HMC contained a thiolated aptamer as its capture probe. Thus, the modification of rinsed Au-NS/CPE was performed by the thiolated aptamer through self-assembly. Subsequently, the electrode modified with aptamer was transferred into a 1 mM 6-mercapto 1-hexanol solution for 60 min. Now, the assembly interface was able to sense the HMC. 

### 2.6. Homocysteine Determination 

To this end, the aptamer-modified CPE (AP/CPE) was immersed in the HMC solution (2 mL) with variable PBS concentrations (0.1 M PB, 0.1 M NaCl, and pH = 7.0) for 60 min. After washing, the electrochemical detection was applied to agglomerated HMC in a 25 mL buffer (0.1 M PB, 0.1 M NaCl, and pH 7.0), exploiting the differential pulse voltammetry (DPV). 

## 3. Results and Discussion

### 3.1. Structure and Morphology

**Figure 1:**
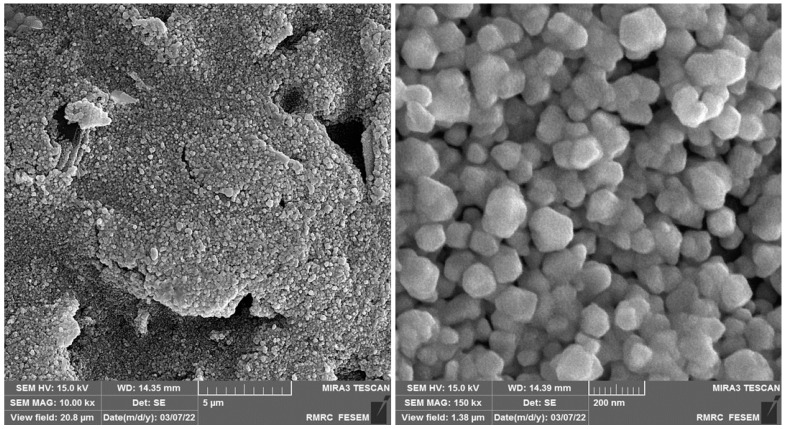
FE-SEM images of Au-NS modified CPE at different magnifications.

The FE-SEM images of Au-NS modified CPE at different magnifications are shown in Figure 1.

### 3.2. Experimental Optimization 

The AP/Au-NS/CPE sensor was examined for its behaviors in terms of buffer concentration, aptamer concentration, type of buffer, and time of interaction, followed by their optimization in a solution containing HMC (0.1 μM).

**Figure 2:**
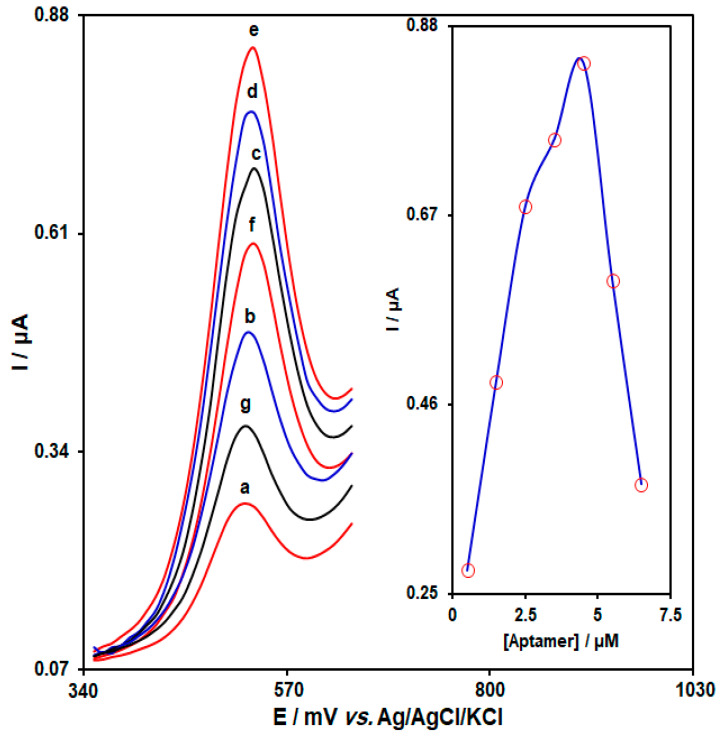
DPVs captured from Au-NS/CPE in PBS (0.1 M, pH = 7.0) with variable aptamer concentrations ((a) 0.5, (b) 1.5, (c) 2.5, (d) 3.5, (e) 4.5, (f) 5.5, and (g) 6.5 μM of aptamer). Inset: plot of peak current versus aptamer concentration, which ranged from 0.5 to 6.5 μM.

Figure 2 illustrates that the current (0.1 µM HMC spiked in 0.1 M PBS (pH = 7.0)) increased along with the Apt concentration and arrived at a bottleneck at 4.5 μM, indicating that the aptasensor was approaching its saturation limit for the Apt concentration. Also, increasing aptamers further could lead to an increase in steric hindrance [48]. Thus, an Apt concentration of 4.5 μM was set as the optimal value.

**Figure 3:**
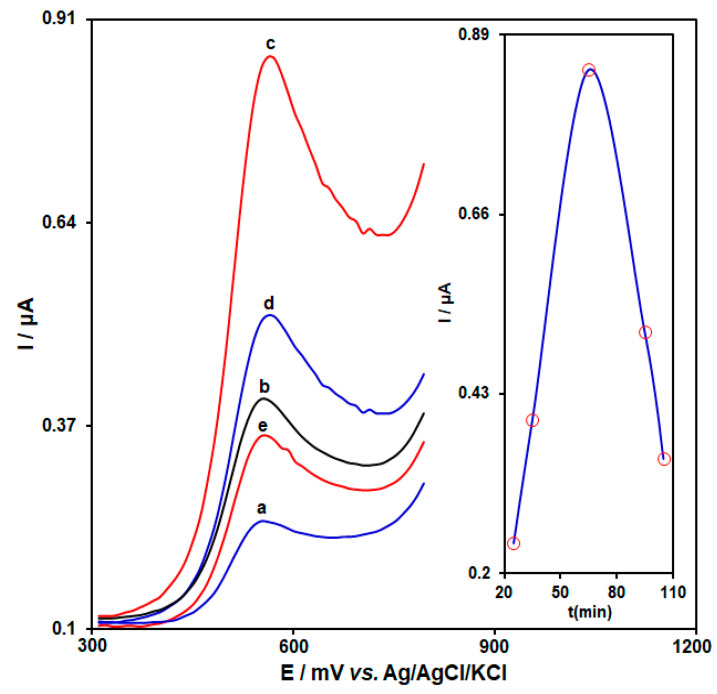
DPVs captured from AP/Au-NS/CPE with an aptamer concentration of 4.5 μM in PBS (0.1 M, pH = 7.0) and variable interaction times of the aptemer with homocysteine ((a) 25, (b) 35, (c) 65, (d) 95, and (e) 105 min). Inset: a plot of peak current versus interaction times ranged from 25 to 105 min.

Figure 3 shows the effect of interaction time (ranging from 25 to 105 min) on the HMC electrochemical signal (0.1 µM HMC spiked in 0.1 M PBS (pH = 7.0)). As shown in Figure 3, the current increased with the increasing of interaction time and reached the maximum current at 65 min. Therefore, 65 min was selected as the optimal interaction time. This trend indicated that the binding sites between aptamer and HMC were approximately saturated after 65 min [49].

**Figure 4:**
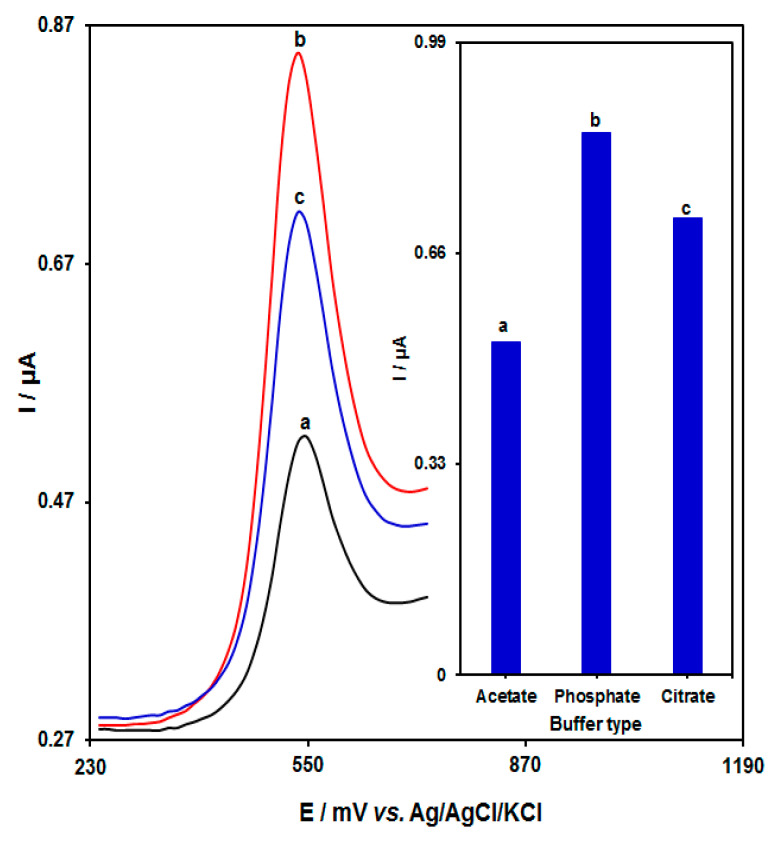
DPVs captured from AP/Au-NS/CPE with an aptamer concentration of 4.5 μM, interaction time of the aptemer with homocysteine of 65 min and various buffers. a–c: (a) acetate, (b) phosphate, and (c) citrate, sequentially. Inset: a plot of peak current versus various buffers.

In Figure 4, the impact of buffer type (citrate, phosphate, and acetate) on HMC detection was explored in solutions carrying 0.1 µM HMC. A sharp increase could be seen in the response of the electrode at 0.1 M PBS (pH = 7.0). Therefore, PBS was selected as the optimal buffer type.

**Figure 5:**
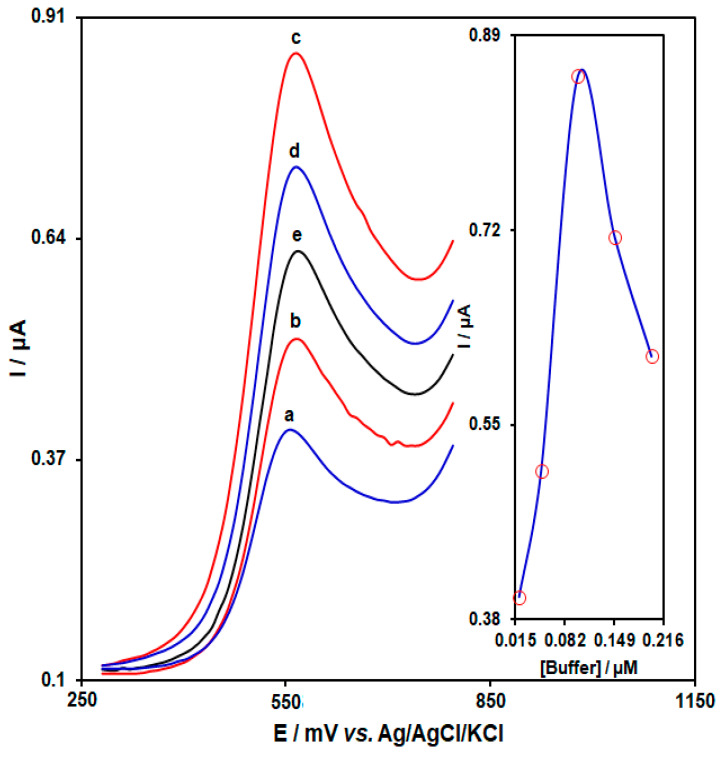
DPVs captured from AP/Au-NS/CPE with the aptamer concentration of 4.5 μM, interaction time of the aptemer with homocysteine of 65 min in PBS (pH 7.0) with variable phosphate buffer concentrations. a–c: (a) 0.02, (b) 0.05, (c) 0.1, (d) 0.15, and (e) 0.2 M, sequentially. Inset: plot of peak current versus variable phosphate buffer concentrations.

The impact of PBS concentration (ranging from 0.02 to 0.2 M) on HMC detection was explored in solutions carrying 0.1 µM HMC spiked in PBS, the results of which are shown in Figure 5. A sharp increase could be seen in the electrode response at 0.1 M PBS. In fact, the concentration of electrolyte can affect the interaction of the aptamer and HMC, resulting in enhancement or reduction of the electrostatic interaction between the aptamer and HMC [49].

Under the optimized circumstances, the best accumulation time, the best buffer, and the best buffer concentration were selected to be 65 min, phosphate buffer, and 0.1 M phosphate buffer for future research. 

### 3.3. Standard Curve and Limit of Detection 

**Figure 6:**
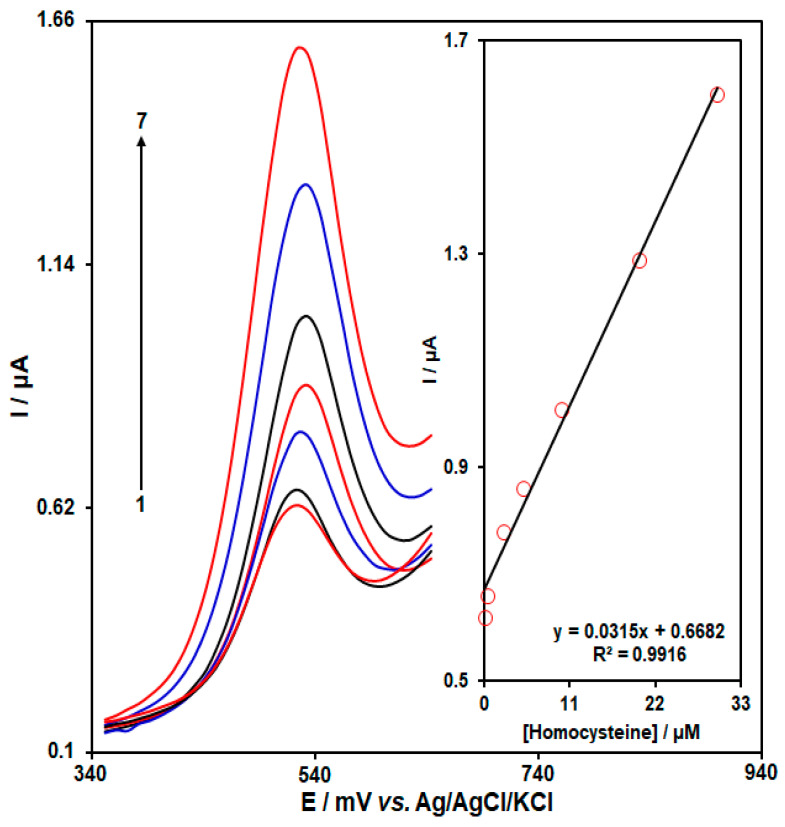
DPVs captured at the aptasensor for homocysteine at variable concentrations in PBS (0.1 M, pH = 7.0). No. 1–7: 0.1, 0.5, 2.5, 5.0, 10.0, 20.0, and 30.0 µM of homocysteine. Inset: standard curve for homocysteine, plot of peak current versus homocysteine concentration.

The standard curve of HMC determination was plotted using as-obtained AP/Au-NS/CPE under optimal circumstances. The solution HMC was detected by its oxidation peak on the modified electrode. Thus, the DPV was recorded for variable HMC concentrations (Figure 6). The peak currents of HMC oxidation on the modified electrode had a linear relationship with HMC concentrations that ranged between 0.1 and 30 μM. The limit of detection (LOD = 3σ) was estimated at 0.03 μM for HMC. 

### 3.4. Real Sample Determination

The practical applicability of an aptasensor was explored by sensing HMC in real specimens of human urine and serum samples in accordance with the standard addition method. The preparation of biological specimens was carried out according to previous reports [50]. The results of the real sample analysis will be accurate when each sample is quickly centrifuged and refrigerated otherwise, glycolysis will increase the HMC content at ambient temperature. Therefore, human serum (1 mL) involves different levels of HMC after isolation of deposited proteins and filtering using a 0.45 μm Millipore filter and diluted with 1 mL of PBS (0.2 M PB, 0.2 M NaCl, pH = 7). Additionally, urine sample (1 mL) was dissolved in 1 mL of PBS for dilution. The urine specimens were refrigerated at 4 °C. Table 1 presents the results of real-sample analysis. Accordingly, the recovery rates were significantly acceptable for the spiked HMC detection, suggesting the appropriateness of the HMC aptasensor for sensing the HMC in biomatrices. 

### 3.5. Comparison of As-Developed Homocysteine Aptasensor with Other Previously Introduced Electrochemical Approaches

A comparison was made for the HMC aptasensor components, by using available electrochemical approaches in terms of accuracy and validation. Clearly, the LOD ranges are better or more linearly proportional to those listed in Table 2. Further, the application of aptamers in the fabrication of the electrode simplifies the detection of the selectivity of the proposed protocol compared to the previous methods (Table 2).

## 4. Conclusions

The current attempt was made to detect the amino acid homocysteine (HMC) using an electrochemical sensor functionalized with aptamers (aptasensor). The proposed aptasensor was examined for its analytical performance under optimized circumstances. It was easy to use and cost-effective. We explored the influences of the concentration of buffer, concentration of aptamer, type of buffer, and time of interaction. The linear dynamic range at a working potential of 520 mV (versus Ag/AgCl) was from 0.1 to 30.0 µM under the optimized circumstances, with the limit of detection as narrow as 0.03 μM. Further, the practical applicability of the sensor was confirmed by sensing HMC in real biological specimens.

## Figures and Tables

**Table 1:** Homocysteine detection in human urine and serum matrices (*n* = 5 (the measurement of each concentration was repeated five times.)); all concentrations are in µM.

Sample	Spiked	Found	Recovery (%)	R.S.D. (%)
**Urine**	0	0.7 (±0.02)	-	3.2
	6.0	6.6 (±0.1)	98.5	2.2
	8.0	8.8 (±0.1)	101.1	1.8
**Human serum**	0	4.1 (±0.1)	-	2.6
	4.0	8.4 (±0.1)	103.7	1.9
	5.0	9.0 (±0.3)	98.9	3.2

**Table 2:** Comparing the homocysteine aptasensor with existing electrochemical approaches previously reported for homocysteine detection.

Method	Limit of Detection (LOD)	linear Dynamic Range (LDR)	Ref.
**Voltammetry/Square wave voltammetry**	0.08 μM	0.1–210.0 μM	[51]
**Voltammetry/Linear sweep voltammograms**	3.3 μM	5.0–800.0 μM	[52]
**Voltammetry/Differential pulse voltammetry**	0.89 μM	2.5–1000.0 μM	[53]
**Voltammetry/Differential pulse voltammetry**	0.15 μM	0.5–900.0 μM	[54]
**Voltammetry/Differential pulse voltammetry**	1.0 μM	1.0–100.0 μM	[55]
**Voltammetry/Differential pulse voltammetry**	0.03 μM	0.1–30.0 μM	This work

## Data Availability

All the data are presented in the manuscript.
